# Novel mechanisms and transcription factors associated with the therapeutic effect of Jakyakgamcho-tang on neuropathic pain

**DOI:** 10.1016/j.gendis.2025.101805

**Published:** 2025-08-12

**Authors:** Su-Jin Baek, Musun Park, A Yeong Lee, Jin-Mu Yi, Sang-Min Park, Seongwon Cha, No Soo Kim

**Affiliations:** a Korean Medicine Data Division, Korea Institute of Oriental Medicine, Daejeon 34054, South Korea; b Korean Medicine Convergence Research Division, Korea Institute of Oriental Medicine, Daejeon 34054, South Korea; c College of Pharmacy, Chungnam National University, Daejeon 34134, South Korea

Neuropathic pain (NP) is a chronic condition with complex molecular mechanisms. Jakyakgamcho-tang (JGT) is derived from Paeoniae Radix and Glycyrrhizae Radix et Rhizoma, and shows a significant therapeutic potential for pain management,^1^ but its underlying mechanisms remain unclear. This study aimed to elucidate the mechanism of action of JGT in NP by systematically investigating the key NP-associated transcription factors (TFs) and signaling pathways regulated by JGT and its compounds. Additionally, we analyzed the transcriptome of JGT-treated PC12 cells and performed *in vitro* experiments to confirm the regulation of target genes.

We identified the key NP-associated genes by performing differentially expressed gene analysis using two public RNA-sequencing datasets (GSE53861^2^ and GSE102937^3^; Table S1). Both NP models showed up-regulation of most differentially expressed genes, and functional enrichment analysis helped identify the key pathways involved in NP pathogenesis (Fig. S1; Table S2). Next, we analyzed the TFs that bind to the promoter regions of the differentially expressed genes in each dataset (Table S3). Comparative analysis showed that Fos and Jun were the common TFs in NP (Fig. 1A). We identified the TFs that bound to the active regions in the NP models by analyzing the H3K4me1 chromatin immunoprecipitation-sequencing data from a public database. We identified 1296 active regions in the NP model (Fig. 1B), and a subsequent motif analysis confirmed Fos as a significant TF that binds to these active regions (Fig. 1C; Table S4). This suggests that Fos is a key TF that mediates NP-associated molecular mechanisms.

**Figure 1:**
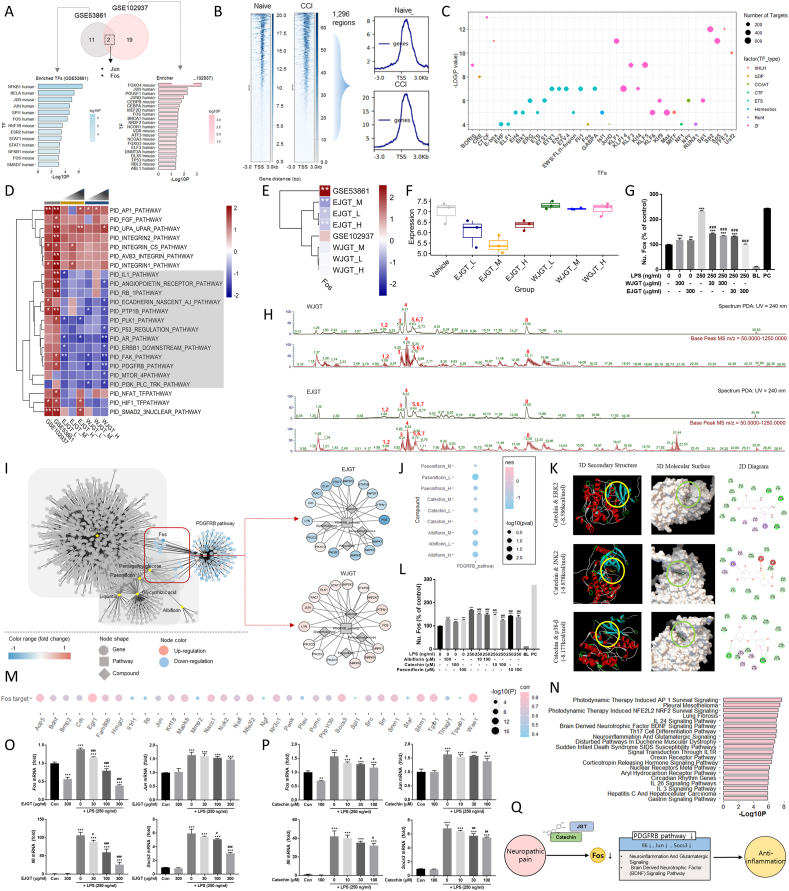
Therapeutic effects of Jakyakgamcho-tang (JGT) and catechin in neuropathic pain via regulation of PDGFRB pathway and Fos. **(A)** Comparison of transcription factor (TF) analysis of differentially expressed gene (DEG) regulation in the two datasets. The Venn diagram in the top panel shows the number of TFs enriched in each dataset (GSE53861 and GSE102937). The diagram highlights the overlap and unique TFs identified in each dataset. The bottom panel shows the detailed TF analysis for each dataset and specific factors that regulate the DEGs in the context of NP. **(B)** Identification of active chromatin regions in the NP model using H3K4me1 chromatin immunoprecipitation-sequencing data. Transcription start site (TSS) heatmap (left panel) shows the chromatin immunoprecipitation signal for H3K4me1 at various distances from the TSSs in both naive and chronic constriction injury (CCI) conditions. The heatmap highlights differences in H3K4me1 enrichment and reflects changes in chromatin accessibility and active enhancer regions. The line plot on the right panel shows the average H3K4me1 chromatin immunoprecipitation signal for naive and CCI samples, which further emphasizes the NP-associated differential chromatin modifications. **(C)** Enriched TF motifs of the identified 1263 active chromatin regions. **(D)** Molecular pathways and processes modulated by JGT treatment. The gray-highlighted regions indicate the specific pathways where expression levels are modulated by JGT and provide insights into the therapeutic effects of JGT in reversing or modulating the NP-associated dysregulated pathways. High dose (H): 100 μg/mL; medium dose (M): 20 μg/mL; and low dose (L): 4 μg/mL ∗*P* < 0.05 and ∗∗*P* < 0.01. **(E, F)** The heatmap (E) and boxplot (F) showing the changes in gene expression of Fos regulated by ethanol and water extracts of JGT (EJGT and WJGT). **(G)** Experimental validation of Fos expression activity. **(H)** Ultraviolet chromatogram (UV = 240 nm), base peak chromatogram (BPC; *m*/*z* = 50–1250), and BPC for each compound (WJGT, upper panel; EJGT, lower panel). **(I)** Compound–target interactive network for six JGT compounds and PDGFRB pathway genes. This network shows the interactions between the six JGT compounds, their known molecular targets, and JGT-modulated PDGFRB pathway genes. The compounds included in the analysis were paeoniflorin, albiflorin, catechin, liquiritin, glycyrrhizic acid, and pentagalloylglucose. **(J)** Expression changes induced by three JGT compounds in the PDGFRB pathway in A549 cells. This figure shows the changes in PDGFRB pathway expression in A549 lung cancer cells following treatment with the three JGT compounds. **(K)** Molecular docking analysis of catechin with mitogen-activated protein kinases (ERK2, JNK2, and p38) involved in Fos expression. The yellow and green circles indicate optimal sites for the interaction of catechin with proteins. **(L)** Experimental validation of anti-inflammatory effects of three JGT compounds. **(M)** Identification of Fos targets through correlation analysis. Correlation coefficients were calculated to determine the relationship between Fos and potential target genes. Strongly correlated genes were selected based on statistical significance (*P* < 0.05) and correlation thresholds. **(N)** Functional enrichment analysis of genes that were significantly associated with Fos expression in correlation analysis. **(O)** Experimental validation of Fos target genes altered by EJGT treatment. Gene expression levels were analyzed using quantitative real-time PCR. **(P)** Experimental validation of Fos target genes altered by catechin treatment: Expression changes of four Fos target genes in response to catechin treatment. **(Q)** The schematic summarizes the key study findings by depicting the proposed molecular mechanisms, key target genes, signaling pathways, and effects of JGT or its active compound.

To investigate the key molecular mechanisms of JGT, we generated transcriptome data from PC12 cells treated with water and ethanol extracts of JGT (WJGT/EJGT) for 24 h at low (4 μg/mL), medium (20 μg/mL), and high (100 μg/mL) concentrations that were confirmed to be non-cytotoxic (Fig. S2). Gene Set Enrichment Analysis (GSEA) was performed using the PID gene sets to identify the shared JGT-modulated molecular pathways. In total, 23 gene sets were identified, and at least one condition showed a significance of *P* < 0.05 (Fig. 1D; Table S5). Furthermore, we investigated the pharmacological mechanisms of JGT by analyzing the expression changes in NP models. We identified 12 pathways that were up-regulated in the NP model and at least one pathway that was significantly down-regulated by JGT treatment. Figure S3 shows the expression networks of the 12 pathways and genes, highlighting Fos as the key TF in the NP model, and the “PDGFRB pathway” and “ERBB1 downstream pathway” as the major Fos-related pathways. Notably, the PDGFRB pathway activates signaling pathways that enhance neuronal excitability and hypersensitivity in damaged nerves,^4^ thereby amplifying the pain signals following nerve injury. In fact, Fos is a key TF linked to these pathways. GSEA and heatmap analyses showed that JGT reduced the expression of PDGFRB pathway genes that were up-regulated in the NP model (Fig. S4), suggesting a role in modulating pain-related signaling. Among these pathway genes, Fos was significantly up-regulated in the NP models; however, EJGT treatment significantly down-regulated Fos expression in PC12 cells (Fig. 1E and F). We confirmed the JGT-mediated Fos regulation by determining the nuclear Fos activity in BV2 microglial cells treated with lipopolysaccharide (LPS) used to induce Fos-mediated inflammation. As shown in Figure 1G, nuclear Fos level increased following LPS treatment for 8 h. However, LPS-mediated nuclear Fos activation was efficiently inhibited by EJGT pretreatment, suggesting that Fos is a key TF in the NP model and an important target for alleviating NP.

Next, we identified the compounds in JGT extracts by performing compound pattern analysis of WJGT/EJGT using ultra-high-performance liquid chromatography coupled with hybrid quadrupole-orbitrap tandem mass spectrometry. The top 10 peaks were determined based on the base peak chromatogram peak area ratio. Among these, eight components were identified by comparison with standard components (Fig. 1H; Fig. S5). We selected the six major compounds of JGT (albiflorin, catechin, glycyrrhizic acid, liquiritin, paeoniflorin, and pentagalloylglucose) based on target gene and disease information from PubChem. To identify key interactions with the PDGFRB pathway, we constructed a compound–target network and integrated it with the JGT-modulated PDGFRB pathway (Fig. 1I). Then, we analyzed transcriptome data to assess the expression profiles of three previously studied compounds in A549 cells.^5^ The normalized enrichment score was negative for the PDGFRB pathway for three JGT compounds: albiflorin, catechin, and paeoniflorin (Fig. 1J). Most PDGFRB pathway genes, including Fos, were down-regulated in A549 cells after treatment with the three compounds (Fig. S6). Additionally, we elucidated the relationship between Fos and these compounds through docking analysis and examining their interactions with ERK2, JNK2, and p38, which are the upstream mitogen-activated protein kinases of Fos (Fig. 1K; Table S6). We found that JNK2 (−8.878 kcal/mol) showed the highest interaction potential with catechin (Table S7). Furthermore, we investigated whether albiflorin, catechin, and paeoniflorin down-regulated LPS-induced Fos activation in BV2 cells and found that all three compounds marginally induced nuclear Fos activation in the absence of LPS treatment (Fig. 1L). However, under LPS-induced inflammatory conditions, pretreatment with all three components inhibited nuclear Fos activation, and catechin showed the most pronounced effect in down-regulating Fos activation in LPS-challenged BV2 cells.

Next, we identified the downstream targets of Fos by performing a correlation analysis between Fos and its target gene expression. In total, 256 Fos target genes were derived from TargetScan, and correlation analysis identified 34 genes that were significantly co-expressed with Fos (*P* < 0.05; Fig. 1M; Table S8). We identified the top 20 most significant pathways by performing GSEA of the 34 Fos target genes (Fig. 1N; Table S9). Fos expression significantly and positively correlated with the expression of three target genes (*Socs3*, *Il6*, and *Jun*; Fig. S7). We confirmed the changes in the expression of Fos and its target genes in response to JGT by measuring their intracellular mRNA levels in BV2 cells co-treated with EJGT and LPS. As shown in Figure 1O, EJGT down-regulated Fos expression in BV2 cells even without LPS challenge. Furthermore, LPS treatment up-regulated Fos expression, which was inhibited in a dose-dependent manner by EJGT pretreatment. Potential Fos target genes, such as *Jun*, *Il6*, and *Socs3*, were up-regulated by LPS treatment, which was inhibited by pretreatment with EJGT except for *Jun*. Next, we determined the effects of catechin on the expression of Fos and its target genes in LPS-treated BV2 cells. Catechin pretreatment marginally but significantly inhibited the expression of Fos and its target genes in LPS-challenged BV2 cells in a dose-dependent manner (Fig. 1P). Figure 1Q summarizes the key TFs involved in NP, along with the underlying mechanisms of JGT and their key compounds in NP.

In conclusion, this study shows that JGT and its key compound catechin inhibit the expression of Fos, which is a key TF involved in NP, by inducing anti-inflammatory effects via modulating the PDGFRB pathway and down-regulating the inflammation-related Fos downstream targets such as *Il6*, *Jun*, and *Socs3*.

## CRediT authorship contribution statement

**Su-Jin Baek:** Writing – original draft, Investigation, Conceptualization, Writing – review & editing, Visualization, Data curation. **Musun Park:** Visualization, Formal analysis, Writing – original draft, Methodology. **A Yeong Lee:** Writing – original draft, Methodology, Conceptualization, Visualization, Formal analysis. **Jin-Mu Yi:** Resources, Validation, Methodology. **Sang-Min Park:** Writing – review & editing, Data curation, Investigation. **Seongwon Cha:** Writing – review & editing, Project administration, Writing – original draft, Funding acquisition. **No Soo Kim:** Writing – original draft, Validation, Writing – review & editing, Visualization, Methodology.

## Funding

This study was funded by the research program of the 10.13039/501100003718Korea Institute of Oriental Medicine (KIOM, No. KSN1739122), Daejeon, South Korea.

## Conflict of interests

The authors declared no conflict of interests.
