# Supplementary material for: Novel mechanisms and transcription factors associated with the therapeutic effect of Jakyakgamcho-tang on neuropathic pain
Source: Genes Dis. 2025 Aug 12;13(4):101805. doi: 10.1016/j.gendis.2025.101805 (PMC13049572; doi:10.1016/j.gendis.2025.101805)
Supplement: Multimedia component 1 [file mmc1.docx]

| **Novel mechanisms and transcription factors associated with therapeutic effect of Jakyakgamcho-tang on neuropathic pain** |
| --- |

**1.Supplementary Figures (7)**

Figure S1. Fos is a major transcription factor in the pathogenesis of neuropathic pain (NP).

Figure S2. WST assay and morphological changes in PC12 cells treated with WJGT and EJGT

Figure S3. Integrated networks among pathways and genes included in gray box are regulated by JGT.

Figure S4. Heatmap of expression changes in PDGFRB pathway and genes

Figure S5. UV chromatogram (UV = 240 nm), base peak chromatogram (BPC; m/z = 50 – 1250), and BPC for each component

Figure S6. Heatmap of changes in PDGFRB pathway expression in A549 cells induced by three JGT compounds

Figure S7. Scatter plot for three Fos target genes.

**2.Supplementary Tables (9): Supplementary Tables.xlsx**

Table S1. List of DEGs in GSE53861 and GSE102937

Table S2. Functional classification of DEGs in GSE53861 and GSE102937

Table S3. Enriched TFs in GSE53861 and GSE102937

Table S4. Enriched Motifs in active regions (H3K4me1)

Table S5. Enriched molecular pathways by JGT in PC12 cells

Table S6. Docking analysis results of three compounds with Fos-related proteins

Table S7. Binding affinity between catechin and c-Fos related proteins

Table S8. Target genes correlated with Fos expression

Table S9. Enriched pathways of Fos expression-correlated target genes

**3.Materials and Methods**


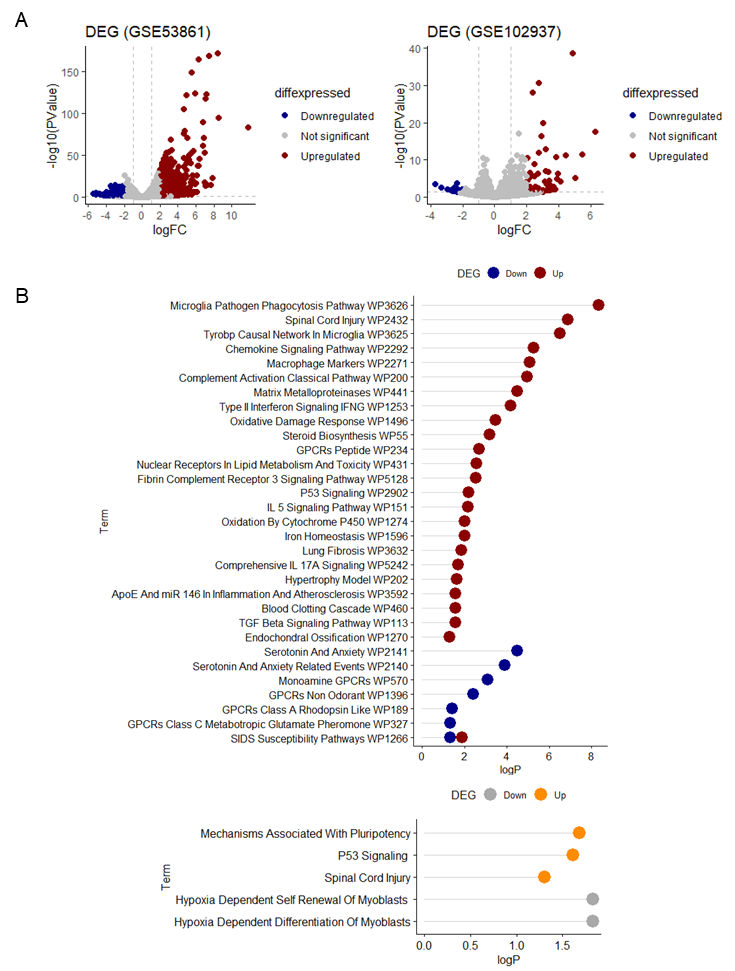


Figure S1. Fos is a major transcription factor in the pathogenesis of neuropathic pain (NP). (A) Differential gene expression in an NP model compared with that in normal control using transcriptome data in Gene Expression Omnibus (GEO) database. Scatter plot showing differentially expressed genes (DEGs) in an NP model across two independent datasets. Each point represents a gene; the x- and y-axes correspond to changes in expression levels (logFC; log2 fold change) and significance (-log10(P value)) in the two datasets. Genes that are significantly upregulated or downregulated in the NP condition are highlighted to emphasize the consistency of the DEG profiles in both datasets. (B) Results of functional enrichment analysis of DEGs identified in (A). Dot plot showing functional enrichment analysis of DEGs from GSE53861 and GSE102937. The top and bottom panels present the functions identified in GSE53861 and GSE102937 datasets, respectively.


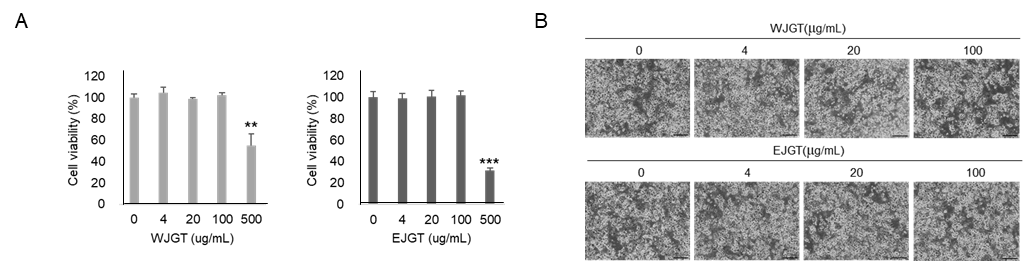


Figure S2. WST assay and morphological changes in PC12 cells treated with WJGT and EJGT. (A) Cell viabilities were measured by WST assay. Relative cell viabilities compared to vehicle-treated control cells are presented as the means ± SD (n=3/group). (B) PC12 cells were treated with 4, 20, 100 μg/mL of WJGT and EJGT for 24h and observed under an inverted microscope (100x magnification). ***p* < 0.01 and * ***p* < 0.001

Figure S2. UV chromatogram (UV = 240 nm), base peak chromatogram (BPC; m/z = 50 – 1250), and BPC for each component: water extract (A), 70% EtOH extract (B) and chemical structures of components (C)


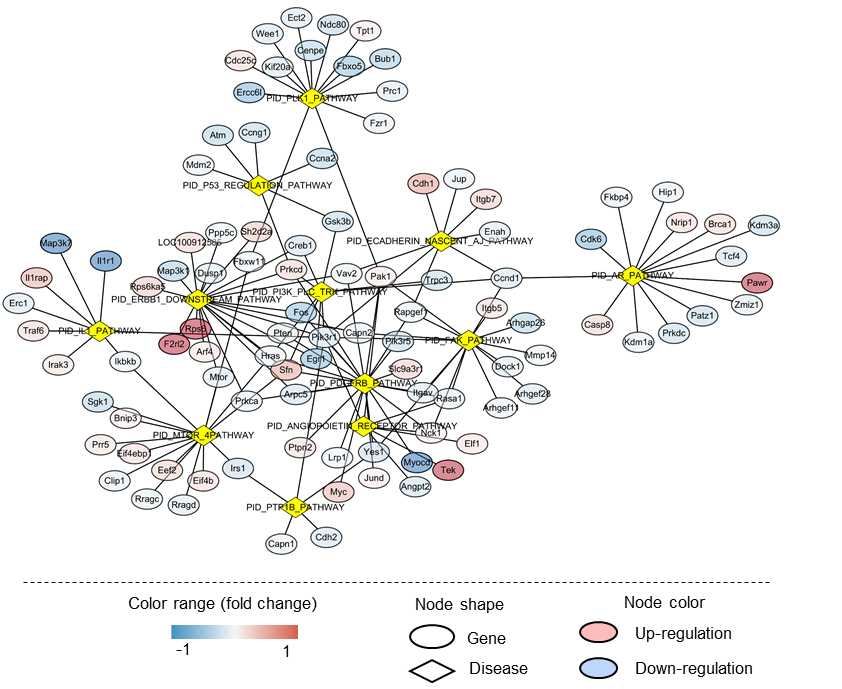


Figure S3. Integrated networks among pathways and genes included in gray box are regulated by JGT.


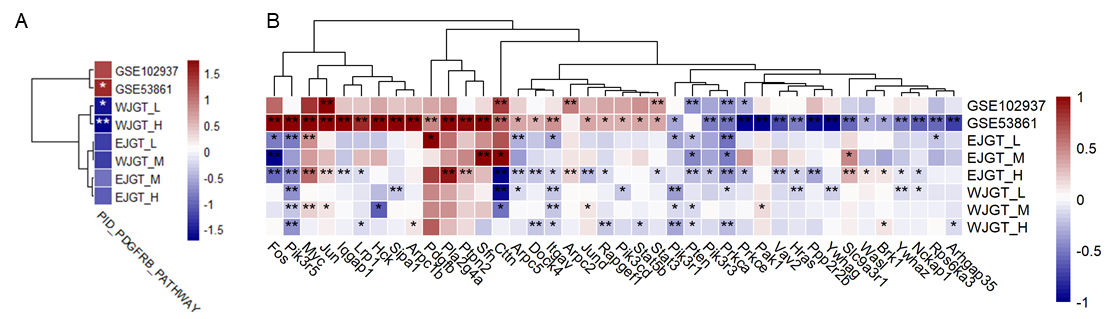


Figure S4. Heatmap of expression changes in PDGFRB pathway and genes. (A)Heatmap of expression changes in PDGFRB pathway. *p < 0.05 and * *p < 0.01 (B) Expression patterns of significantly modulated genes of the PDGFRB pathway. *p < 0.05 and * *p < 0.01.


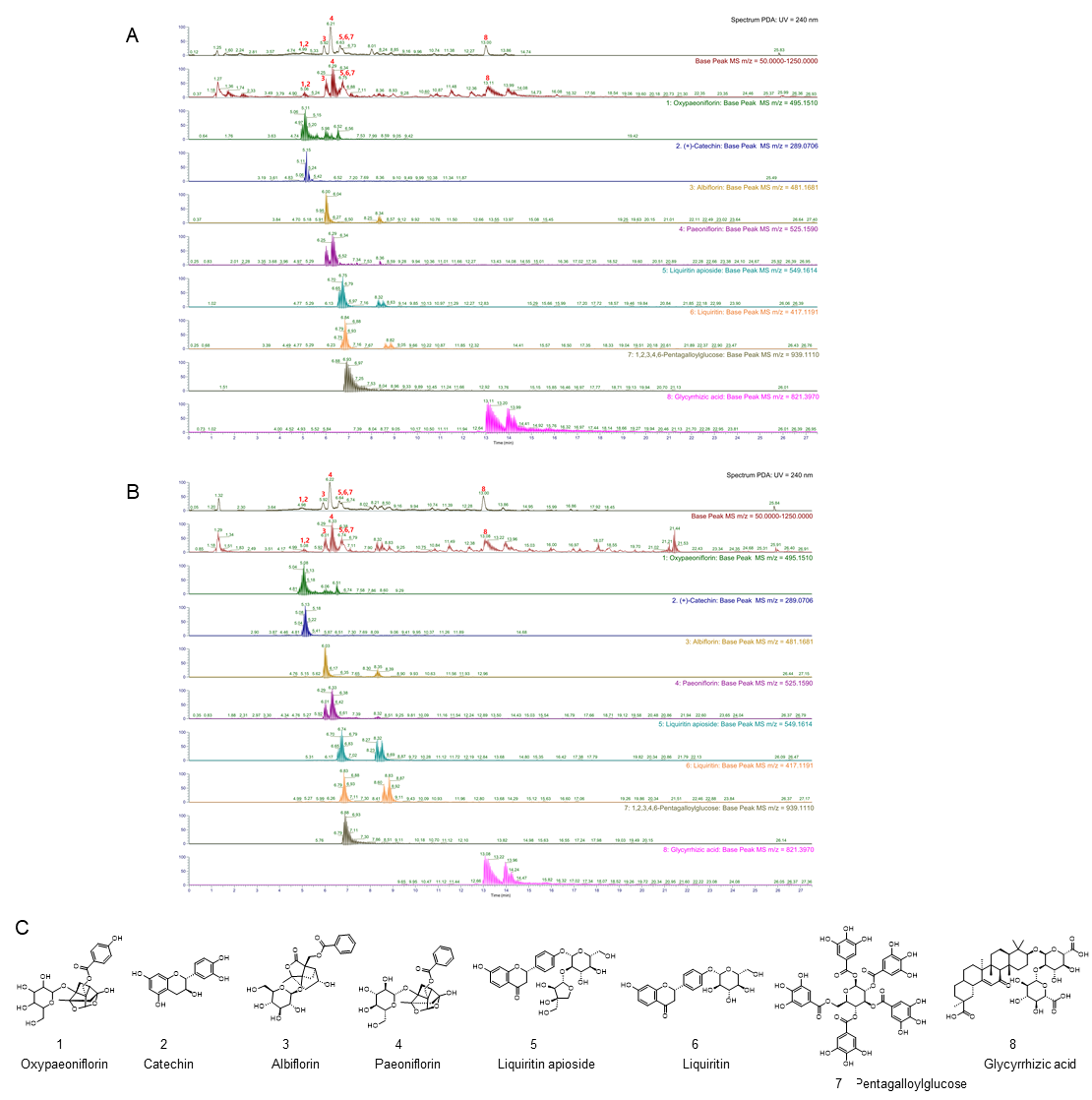


Figure S5. UV chromatogram (UV = 240 nm), base peak chromatogram (BPC; m/z = 50 – 1250), and BPC for each component in water extract of JGT (A), ethanol extract of JGT (B), and chemical structures of components (C).


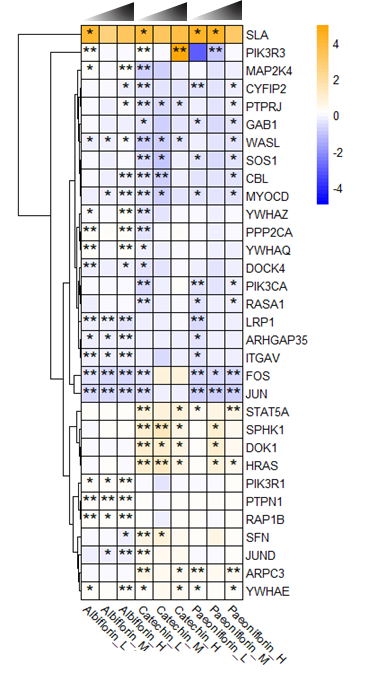


Figure S6. Heatmap of changes in PDGFRB pathway expression in A549 cells induced by three JGT compounds. color scale represents relative expression levels with yellow indicating upregulation and blue indicating downregulation compared with that in the control group.


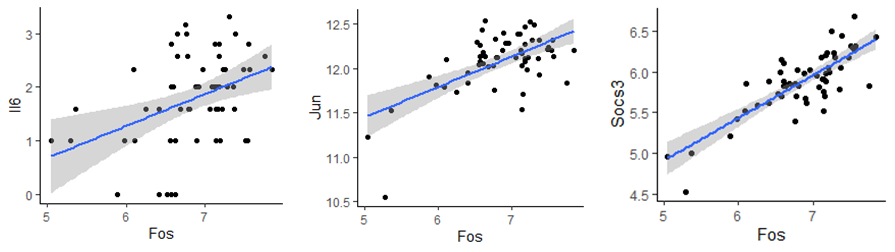


Figure S7. Scatter plot for three Fos target genes. Scatter plots showing the correlation between Fos expression and three identified target genes.

**Materials and methods**

**Chemicals**

Albiflorin (A0477, CAS No. 39011-90-0, 95.7% purity**)**, catechin (A0158, CAS No. 225937-10-0, 99.7% purity**)**, and paeoniflorin (A0133, CAS No. 23180-57-6, 98.7% purity) were purchased from Chengdu MUST Bio-Technology Co., Ltd. (Chengdu, China). Dimethyl sulfoxide (DMSO; D8418) and lipopolysaccharide (LPS, L2654) were purchased from Sigma-Aldrich (St. Louis, MO, USA).

**Plant materials and preparation of WJGT and EJGT**

Paeoniae Radix and Glycyrrhizae Radix et Rhizoma, which were prepared in compliance with the Korean Pharmacopoeia, were obtained commercially from Kwangmyungdang Co. (Ulsan, Republic of Korea). The morphological and genetic identification of the plant materials were authenticated by Dr. Goya Choi, Herbal Medicine Resources Research Center, Naju, Republic of Korea. Voucher specimens of *Paeoniae* Radix (#2-21-0001) and *Glycyrrhizae* Radix et Rhizoma (#2-21-002) were deposited in the Korean Herbarium of Standard Herbal Resources, Herbal Medicine Resources Research Center, Korea Institute of Oriental Medicine. An aqueous extract of JGT (WJGT) was prepared as described previously 16. Aquo-ethanolic extract of JGT (EJGT) was prepared as follows: equal quantities of dried *Paeoniae* Radix (450 g) and *Glycyrrhizae* Radix et Rhizoma (450 g) powders were resuspended in four times the volume of 70% ethanol and sonicated for 1 h. The extraction was repeated using the same volume of fresh 70% ethanol. The two extract solutions were combined, filtered through a 53-μm mesh filter, vacuum-concentrated at 60 °C, and lyophilized in a freeze-dryer (LP-20, Ilshin-Bio-Base, Dongduchen, Republic of Korea) to obtain final WJGT and EJGT extracts with 24.4% and 19.5% yields, respectively. WJGT and EJGT stock solutions were prepared in 2% DMSO/phosphate-buffered saline (PBS, #10010-023, Thermo Fisher Scientific, Rockford, IL, USA) and filtered through a syringe filter with a 0.22-μm regenerated cellulose membrane (#17764-ACK, Satorius, Göttingen, Germany). The sterilized WJGT and EJGT stock solutions were stored at -80 °C until they were used in further experiments.

**UHPLC-MS pattern analysis of JGT**

LC-MS pattern analysis of WJGT and EJGT was performed using an ultrahigh-performance liquid chromatography-Q-Orbitrap-MS (UHPLC-Q-Orbitrap-MS) equipped with a Dionex UltiMate 3000 UHPLC system, Thermo Q-Exactive mass spectrometer (Thermo Fisher Scientific, Waltham, MA, USA) and Acquity BEH C18 (Waters, USA) UHPLC column at a 3.0-μL injection volume. The column and sample temperatures were set to 40 ℃ and 4 ℃, respectively. The mobile phase comprised distilled water containing 0.1% formic acid (A) and acetonitrile (B), which was subjected to the following gradient program: 3% B for 0–1 min, 3–15% B for 1–2 min, 15–50% B for 2–13 min, 50–100% B for 13–20 min, isocratic 100% B for 20–23 min, 100–3% B for 23–23.5 min, and isocratic 3% B for 23.5–27.5 min. The Q-Orbitrap-MS conditions were as follows: positive and negative modes of ESI ion source, full MS-ddMS2 scan mode, 100–1500 m/z scan range, 320 ℃ capillary temperature, 3.8 kV spray voltage, 10 au of AUX gas, 40 au of Sheath gas, 70,000 MS resolution, and 17,500 MS/MS resolution. Xcalibur v. 3.0 and Tracefinder v. 3.2 software were used for LC-MS analysis.

All peaks that were detected using LC-MS were searched in the in-house library using Tracefinder ver. 3.2, and all eight major peaks were confirmed using standard components. They were oxypaeoniflorin (≥ 98.37%; MedChemExpress, NJ, USA), (+)-catechin (USP reference standard; Merck KGaA, Darmstadt, Germany), albiflorin (≥ 99.0%; Chengdu Must Bio-Technology Co., Ltd, Chengdu, China), paeoniflorin (≥ 98.0%; Sigma-Aldrich, Merck KGaA, Darmstadt, Germany), liquiritin apioside (≥ 98.0%; ChemFaces, Hubei, China), 1,2,3,4,6-pentagalloylglucose (≥ 98.0%; ChemFaces, Hubei, China), liquiritin (≥ 99.0%; Chengdu Must Bio-Technology Co., Ltd, Chengdu, China), and glycyrrhizic acid (≥98.0%; ChemFaces, Hubei, China).

**PC12 cell culture and cell viability**

The rat adrenal gland pheochromocytoma cell line PC12 was obtained from the American Type Culture Collection (CRL-1721, Manassas, VA, USA). The PC12 cells were maintained in a conventional plastic cell culture ware containing a growth medium comprising DMEM with 4.5 g/L glucose (#11995-065) basal medium supplemented with 10% heat-inactivated horse serum (HI-HS, #26050-088), 5% non-heat inactivated fetal bovine serum (nHI-FBS, #16000-044), and 1% penicillin/streptomycin (Pen/Strep, #15140-122). The cells were cultured at 37 °C in a humidified air containing 5% CO_2_. The murine microglial cell line BV2 was provided by Dr. M. Y. Lee (Korea Institute of Oriental Medicine). The BV2 cells were maintained in DMEM supplemented with 10% FBS and 1% Pen/Strep in a 5% CO_2_-balanced humidified incubator at 37 °C. All basal media and cell culture supplements were obtained from Thermo Fisher Scientific (Waltham, MA, USA).

The viability of the PC12 cells was assessed using the WST-8 cell Viability Assay Kit (QM2500; Biomax, Guri, Republic of Korea) to determine the maximum tolerable WJGT and EJGT concentrations for RNA sequencing. PC12 cells were plated at a density of 2 ×10^4^ cells/well in a 96-well cell culture plate precoated with collagen type IV (#354429, Corning, NY, USA). The cells were neuronally differentiated for 4 days in a differentiation medium (DM) comprising a DMEM basal medium supplemented with 100 ng/mL recombinant rat β-nerve growth factor (#556-NG-100, R&D Systems, Minneapolis, MN, USA), 1% N-2 plus media supplement (AR003, R&D systems), 0.5% FBS, and Pen/Strep. Next, the DM was replaced with fresh DM containing serially diluted WJGT or EJGT. The final DMSO concentration was adjusted to 0.1% for all treated cells. After 24 h, the cells were washed once with PBS and incubated with 100 μL DM containing 10% WST-8 stock solution from the kit. The viable cells developed an orange color, which was detected at OD_450_ using a SpectraMax3 Microplate Reader (Molecular Devices, Sunnyvale, CA, USA). The relative cell viability (% of control) was determined by comparing the OD_450_ of WJGT- or EJGT-treated cells with that of the vehicle (0.1% DMSO)-treated cells. The inhibitory concentrations 20 (IC_20_) of WJGT and EJGT in the differentiated PC12 cells were determined using the SoftMax Pro Software (Version 6.4, Molecular Devices).

**Total RNA preparation**

Total RNA was isolated from the cell cultures using QIAzol Lysis Reagent (#79306, Qiagen Science, Germantown, MD, USA) for RNA sequencing or the Easy-Spin^TM^ Total RNA Extraction Kit (#17211, iNtRON Biotechnologies, Seongnam, Republic of Korea) for quantitative PCR (qPCR). Cells treated with WJGT, EJGT, or a combination of drugs were washed twice with ice-cold PBS and subjected to total RNA extraction according to the manufacturer’s instructions. RNA concentration was determined using the Quant-it RiboGreen RNA Assay Kit (R11490; Thermo Fisher Scientific) for RNA sequencing by following the manufacturer’s instructions or using a NanoDrop^TM^ 2000 spectrophotometer (Thermo Fisher Scientific). RNA quality was determined based on two criteria: RNA integrity number (RIN >7) and 28S:18S ribosomal RNA ratio (>1.0), which were evaluated using an Agilent 2100 Bioanalyzer System (Agilent Technologies, Waldbronn, Germany).

**Library preparation and RNA-sequencing**

Total RNA was isolated from the tissues using an easy-spin Total RNA Extraction Kit. Total RNA (1 mg) was processed to prepare an mRNA sequencing library using the MGIEasy RNA Directional Library Prep Kit (MGI-Tech) according to the manufacturer’s instructions. The first step involved the purification of the poly A-containing mRNA molecules using poly T oligo-attached magnetic beads. Next, the mRNA was fragmented into small pieces using divalent cations at elevated temperatures. The cleaved RNA fragments were copied into first-strand cDNA using reverse transcriptase and random primers. Strand specificity was achieved using a reverse transcriptase directional buffer, followed by second-strand cDNA synthesis. A single 'A' base was added to these cDNA fragments, followed by the ligation of the adapter. The products were purified and enriched using PCR to create a final cDNA library. The double-stranded library was quantified using a QuantiFluor ONE dsDNA System (Promega). The library was circularized at 37 °C for 30 min and digested at 37 °C for 30 min, followed by clean-up of the circularization products. To prepare DNA nanoballs (DNB), the library was incubated at 30 °C for 25 min using DNB enzyme. Finally, the library was quantified using a QuantiFluor ssDNA System (Promega). The prepared DNB was sequenced using the MGIseq system (MGI) with 150-bp paired-end reads.

**Data quality control and calculating expression values**

The quality of the raw sequence data was assessed using FastQC, and low-quality bases were trimmed using Trim Galore (https://www. bioinformatics. babraham. ac=/). Next, the reads were aligned with the rat reference genome (Rn6) using STAR aligner (v 2.7.3a)^17^. The read counts and transcripts per million (TPM) were calculated as gene expression abundance using the RSEM software (v 1.3.3) ^18^ and gene annotation rn6 Gene Transfer Format (GTF) file from the UCSC genome browser (https://hgdownload.soe.ucsc.edu/goldenPath/rn6/bigZips/genes/).

**Neuropathic pain RNA-seq data and analysis**

To investigate the changes in the expression of the NP-associated genes, two transcriptome datasets were obtained from the Gene Expression Omnibus (GEO) database:

1) GSE53861 (rat NP model): This dataset was derived from a rat model of NP in which L5 spinal nerve transection (SNT) had been performed. The dorsal root ganglia (DRG) tissues were collected on postoperative day 7 for RNA sequencing. Transcriptomic data were generated using the Illumina Genome Analyzer IIx platform, and six naïve and nine DRG samples were used for expression analysis.

2) GSE102937 (Mouse NP model): This dataset was derived from a mouse spared nerve injury (SNI) model under 2–4% isoflurane anesthesia. Transcriptomic data were obtained from three naïve and nine SNI samples from injured mice. These datasets provide gene expression profiles of rat and mouse DRG tissues after neuropathic injury, which enabled the identification of differentially expressed genes (DEGs) associated with NP. For each dataset, the DEGs were selected using GEO2R by comparing the injured samples with the naïve controls under the conditions: threshold of log2 |fold change| > 1 and p-value < 0.05.

**Enriched transcription factor analysis in NP**

The DEGs from each dataset were analyzed for transcription factor binding site (TFBS) enrichment using the TRRUST database. Enrichment analysis was performed using EnrichR, which predicts TFs based on over-represented TFBS in the promoters of the DEGs. The TFs that were significantly enriched in each dataset were identified based on an adjusted p-value < 0.05 (with multiple testing correction applied using the Benjamini–Hochberg method). The candidate transcription factors were selected for further analysis and interpretation.

**Histone ChIP-seq analysis and Motif analysis**

We used public H3K4me1 ChIP-seq datasets (rat) downloaded from the NCBI GEO database GSE210321 ^19^, which was generated from two naïve and two CCI model rats. Downstream analysis was performed on the regions with increased and decreased accessibility of H3K4me1 regions after nerve injury provided in this dataset. The density plot of the H3K4me1 chip signal was created using deepTools2 ^20^. Motif analysis of the positive region in H3K4me1 was performed using the Homer findMotifsGenome.pl program ^21^, and matches to known motifs were identified. Candidate motifs were considered significant if the q-value was < 5 × 10^-4^ (threshold).

**Gene set enrichment analysis (GSEA)**

GSEA was performed on pre-ranked values established through the DEG analysis using the “fgsea” (v 1.12.0) package in Bioconductor (http://bioconductor.org/packages/fgsea/) and GitHub (https://github.com/ctlab/fgsea/). Gene sets with hallmark and pathway interaction databases (PID) used as inputs were obtained from the Molecular Signature Database (MSigDB). These hallmark pathways provided an overview of the anti-inflammatory effects of JGT. PID pathways were used to analyze a selected set of novel pathways. When identifying the enriched pathways, 28 hallmarks and 25 PID pathways were selected based on their normalized enrichment scores (NES) and at least one condition showing a change of *p* < 0.05.

**Integrated network analysis for small molecules, potential target genes, and PDGFRB pathway genes**

To analyze the effects of JGT phytochemicals, the target genes of six compounds (albiflorin, liquiritigenin, catechin, glycyrrhizic acid, pentagalloylglucose, and paeoniflorin) selected as JGT compounds and JGT genes associated with the PDGFRB pathway were integrated into the JGT compound–target gene network and visualized using Cytoscape. The target genes of the six compounds were selected as active compounds using “chemical-target interactions” provided by PubChem (https://pubchem.ncbi.nlm.nih.gov), and the JGT genes were selected as a set of genes that showed changes in expression in the PDGFRB pathway after JGT treatment.

**Correlation analysis of Fos and its target genes**

The correlation between Fos expression and the expression of all Fos target genes was computed using Pearson’s correlation coefficient (r) depending on the distribution of the data. A correlation matrix was generated, and statistically significant correlations were identified. Genes with a correlation coefficient that were higher than a predefined threshold (e.g., |r| > 0.5) and p-value < 0.05 were selected as potential Fos target genes. Dot and scatter plots were generated for candidate Fos target genes to visualize the correlation between Fos and its putative targets.

**Docking analysis**

The 3D structures of albiflorin, catechin, and paeoniflorin used for docking analysis were obtained from PubChem (accessed on April 25, 2024). Compounds with 3D sdf structures were downloaded from the PubChem Database ^22^ and converted to pdbqt files using the OpenBabel software (Open Babel GUI v 3.1.1) ^23^. To predict the proteins involved in Fos expression, AP-1 TF and its related proteins were selected as docking proteins. The selected AP-1^24^ TF-related proteins were c-Fos and c-Jun (AP-1 subunits) and ERK (ERK1, ERK2), JNK (JNK1, JNK2, JNK3), and p38 (p38-α, p38-β, p38-γ, p38-δ) (MAPKs that directly affect AP-1 in the MAPK signaling pathway) ^25;26^. The 3D protein structure information was obtained from those of human proteins provided in the AlphaFold database (alphafold_v2, <https://ftp.ebi.ac.uk/pub/databases/alphafold/v2/>) ^27^ and converted to pdbqt files using the OpenBabel Python library (v 3.1.0). The proteins were fixed in a rigid form for analyses under constant conditions using identical parameters. Docking analysis between the compounds and proteins was performed using the AutoDock Vina Python library (v 1.2.0) ^28^. The docking parameters were: center=(0,0,0), box size=126, and exhaustiveness=100. The interaction with the optimal affinity value was selected through five independent trials (n_poses=5). Docking analysis was visualized by selecting the interaction with the highest binding potential among catechin and the ERK, JNK, and p38 families and using the Discovery Studio Visualizer software (v 21.1.0.20) to generate the 3D secondary structures and 2D diagrams; AutoDockTools (v 1.5.6) was used to visualize the 3D molecular surface.

**Experimental validation of Fos activity**

Nuclear Fos TF activation in BV2 cells following drug treatment was determined using the c-FOS Transcription Factor Assay Kit (ab207194, Abcam, Cambridge, UK) according to the manufacturer’s instructions with slight modifications. Briefly, the cells were pretreated with WJGT (0–300 μg/mL), EJGT (0–300 μg/mL), or the phytochemicals individually (0–100 μM) for 1 h, followed by exposure to 250 ng/mL of LPS for 8 h. Then, the cells were washed with ice-cold PBS. The nuclear extract was prepared using ice-cold hypotonic buffer (20 mM HEPES [pH 7.5], IBS-BH004, iNtRON Biotechnology, Seongnam, Republic of Korea; 0.1 mM EDTA, #78444, Thermo Fisher Scientific) supplemented with 1% Halt^TM^ protease and phosphatase inhibitor cocktail (#78444, Thermo Fisher Scientific). Protein concentration was determined using a bicinchoninic acid assay (BCA, #23227, Thermo Fisher Scientific). The concentration of the nuclear extracts was adjusted to 1 μg/μL using the nuclear extraction buffer, and an equal quantity of nuclear extract (5 μg) was used to determine nuclear FOS activation by following the manufacturer’s instructions.

**Experimental validation of Fos targets**

Changes in the intracellular mRNA expression of *Fos* target genes in BV2 cells were determined using qPCR. Briefly, the cells were pretreated with EJGT (0–300 μg/mL) or catechin (0–100 μM) for 1 h and exposed to 250 ng/mL of LPS for 8 h. Total RNA was extracted from the cells as described in section 2.5., and total RNA concentration was adjusted to 100 ng/μL using pure water. First-strand cDNA was synthesized from 1 μg of total RNA using a High-Capacity cDNA Reverse Transcription Kit (#4368814, Thermo Fisher Scientific) as per the manufacturer’s instructions, followed by 50-fold dilution with pure water. qPCR was performed using 250 μM of gene-specific primer pairs (Genotech, Daejeon, Republic of Korea) and Power SYBR^TM^ Green PCR Master Mix (#4367659; Thermo Fisher Scientific) in a CFX96^TM^ real-time PCR system (Bio-Rad, Hercules, CA, USA). The expression of each target gene was calculated using the 2-^ΔΔCt^ method and normalized to that of glyceraldehyde 3-phosphate dehydrogenase (*Gapdh*). The relative expression of each target gene was determined by comparison with the expression in the vehicle-treated control cells. Primer sequences for *Fos*, *Jun*, *Il6*, *Socs3*, and *Gapdh* are summarized in Table 1.

**Table 1. Sequence of gene-specific primers for qPCR**

| Target | Forward (5′ → 3′) | Reverse (5′ → 3′) | Ref. sequence |
| --- | --- | --- | --- |
| *Fos* | GGGAATGGTGAAGACCGTGTCA | GCAGCCATCTTATTCCGTTCCC | NM_010234 |
| *Jun* | CAGTCCAGCAATGGGCACATCA | GGAAGCGTGTTCTGGCTATGCA | NM_010591 |
| *Socs3* | GGACCAAGAACCTACGCATCCA | CACCAGCTTGAGTACACAGTCG | NM_007707 |
| *Il6* | GAGGATACCACTCCCAACAGACC | AAGTGCATCATCGTTGTTCATACA | NM_031168 |
| *Gapdh* | AAGGTGGTGAAGCAGGCAT | GGTCCAGGGTTTCTTACTCCT | NM_001001303 |

**Statistical analysis**

All data obtained from the in vitro studies were analyzed using GraphPad Prism (ver. 9.5.1, GraphPad Software, San Diego, CA, USA). The results are presented as the mean ± standard deviation of multiple experiments. The difference in mean among the groups was evaluated using one-way analysis of variance (ANOVA), followed by Dunnett’s multiple comparison test. Statistical significance was set at *p* < 0.05.

**Data Availability Statement**

The raw and processed expression data have been deposited in the NCBI Gene Expression Omnibus (GEO, https://www.ncbi.nlm.nih.gov/geo/) under the accession number GSE289929 (reviewer access link: https://www.ncbi.nlm.nih.gov/geo/query/acc.cgi?acc=GSE289929; secure token: shohkuesjvidjuf).
